# Paternally Inherited Noonan Syndrome Caused by a *PTPN11* Variant May Exhibit Mild Symptoms: A Case Report and Literature Review

**DOI:** 10.3390/genes15040445

**Published:** 2024-03-31

**Authors:** Ji Yoon Han, Joonhong Park

**Affiliations:** 1 Department of Pediatrics, College of Medicine, The Catholic University of Korea, Seoul 06591, Republic of Korea; han024@catholic.ac.kr; 2 Department of Laboratory Medicine, Jeonbuk National University Medical School and Hospital, Jeonju 54907, Republic of Korea; 3 Research Institute of Clinical Medicine of Jeonbuk National University-Biomedical Research Institute of Jeonbuk National University Hospital, Jeonju 54907, Republic of Korea

**Keywords:** Noonan syndrome, *PTPN11* p.Arg498Trp, paternal inheritance, short stature, intellectual disability, variable expressivity, incomplete penetrance

## Abstract

Background: Noonan syndrome (NS)/Noonan syndrome with multiple lentigines (NSML) is commonly characterized by distinct facial features, a short stature, cardiac problems, and a developmental delay of variable degrees. However, as many as 50% of individuals diagnosed with NS/NSML have a mildly affected parent or relative due to variable expressivity and possibly incomplete penetrance of the disorder, and those who are recognized to have NS only after a diagnosis are established in a more obviously affected index case. Methods: In order to collect intergenerational data reported from previous studies, electronic journal databases containing information on the molecular genetics of *PTPN11* were searched from 2000 to 2022. Results: We present a case of a proband with a *PTPN11* variant (c.1492C > T/p.Arg498Trp) inherited from an asymptomatic father, displaying only mild intellectual disability without classical symptoms of NS. Among our cases and the reported NS cases caused by the *PTPN11* p.Arg498Trp variant, cardiac abnormalities (6/11), facial dysmorphism (7/11), skin pigmentation (4/11), growth problems (4/11), and sensorineural hearing loss (2/11) have been observed. NS/NSML patients with the *PTPN11* p.Arg498Trp variant tend to exhibit relatively lower frequencies of skin pigmentation, facial dysmorphism and cardiac abnormalities and mild symptoms compared to those carrying any other mutated *PTPN11*. Conclusions: Paternally inherited NS/NSML caused by a *PTPN11* p.Arg498Trp variant, including our cases, may exhibit relatively lower frequencies of abnormal features and mild symptoms. This could be ascribed to potential gene–gene interactions, gene–environment interactions, the gender and phenotype of the transmitting parent, or ethnic differences that influence the clinical phenotype.

## 1. Introduction

Noonan syndrome (NS)/Noonan syndrome with multiple lentigines (NSML) is commonly characterized by distinctive facial features, a short stature, cardiac problems, and developmental delay of variable degrees [1]. The molecular diagnosis of NS involves identifying heterozygous pathogenic variants in *BRAF*, *KRAS*, *MAP2K1*, *MRAS*, *NRAS*, *PTPN11*, *RAF1*, *RASA2*, *RIT1*, *RRAS2*, *SOS1*, or *SOS2*, inherited in an autosomal dominant manner [2,3]. Variants associated with NS in SHP2 display elevated phosphatase activity, whereas variants linked to NSML result in reduced catalytic activity [4,5]. Among them, *PTPN11* plays a regulatory role in neurogenesis, influencing processes such as neuronal differentiation, neurite outgrowth and migration, as well as the proliferation and maturation of oligodendrocytes [6,7]. Variants in *PTPN11* may contribute to the manifestation of neurodevelopmental disorders observed in some patients with NS or NSML. However, as many as 50% of individuals diagnosed with NS/NSML have a mildly affected parent or relative due to variable expressivity and possibly incomplete penetrance of the disorder, and those who are recognized to have NS only after a diagnosis are established in a more obviously affected index case. In animal models of NS, variants in *PTPN11* have been associated with deficits in intention, hyperactivity, memory impairment, and learning disabilities [8,9]. Such alterations in brain development can lead to developmental deficits associated with NS/NSML. Variants in *PTPN11* are responsible for RASopathies, including NS and NSML, with a prevalence of up to 50% [10]. To date, several missense variants of *PTPN11* have been reported, primarily situated in the PTP domain, demonstrating a dominant negative effect: p. Tyr279Cys/Ser, p.Ala461Thr/Ser, p.G464A, p.Thr468Met/Pro, p.Arg498Leu/Trp, p.Gln506Pro, and p.Gln510Glu/Gly [11]. Two of these variants (p.Trp279Cys and p.Thr468Met) are prevalent, accounting for about two-thirds of cases.

In this context, we present a case of a proband with a *PTPN11* variant (c.1492C>T/p.Arg498Trp) inherited from an asymptomatic father, displaying only mild intellectual disability (ID) without other associated symptoms of RASopathy. The *PTPN11* p.Arg498Trp variant has previously been reported in patients with NS/NSML, exhibiting a spectrum of clinical features, including multiple lentigines, cardiac abnormalities, facial dysmorphism, and variable degrees of cognitive impairments [12,13,14]. The collective phenotypes reported in previous studies, including our case, contribute to an enhanced understanding of *PTPN11* variants. No single clinical feature was shown across the previous reports; however, this family revealed few symptoms contributing to an enhanced understanding of *PTPN11* variants.

## 2. Materials and Methods

### 2.1. Genetic Testing

Considering the short stature and ID observed in the proband, we pursued sequential genetic testing for various familial short stature and/or ID disorders. These included conventional karyotyping, an *FMR1* (CGG)n triplet repeat test, and chromosomal microarray analysis. However, no pathogenic alterations were identified in these initial genetic tests. Subsequently, we employed a clinical exome sequencing utilizing the Celemics G-Mendeliome Clinical Exome Sequencing Panel (Celemics, Inc., Seoul, Republic of Korea), which includes a wide range of 7000 genes associated with clinically significant Mendelian genetic diseases, even including all clinically significant regions. Massively parallel sequencing was carried out with the DNBSEQ-G400RS High-throughput Sequencing Set and DNBSEQ-G400 sequencer (MGI Tech Co. Ltd., Shenzhen, China). The interpretation of pathogenic variants followed the standards and guidelines set by the American College of Medical Genetics and Genomics (ACMG) and the Association for Molecular Pathology (AMP).

### 2.2. Literature Review of PTPN11 p.Arg498Trp

In order to collect intergenerational data reported from previous studies, electronic journal databases, including KoreaMed (http://koreamed.org, accessed on 10 November 2023) and PubMed (https://www.ncbi.nlm.nih.gov/pubmed, accessed on 10 November 2023), containing information on the molecular genetics of *PTPN11*, were searched from 2000 to 2022. The following terms were used in search strategies: *PTPN11* mutation, p.Arg498Trp, Rasopathies, LEOPARD syndrome, and Noonan syndrome. All related citations were retrieved to find other relevant articles that were not identified in the initial research. The literature search included Korean as well as English articles. Only reported cases with well-documented relevant information about the gender, variant type of the *PTPN11* gene, and/or the detailed phenotypes of the transmitting parent and the affected child were included. After a literature search related to NS/NSML with *PTPN11* p.Arg498Trp [12,13,15,16,17,18,19], 39 parent–child matched pairs with information about each gender, the p.Arg498Trp of the *PTPN11* gene, and/or the NS/NSML’s clinical phenotypes were included in this study.

## 3. Patient Description

### 3.1. The Proband

**Figure 1:**
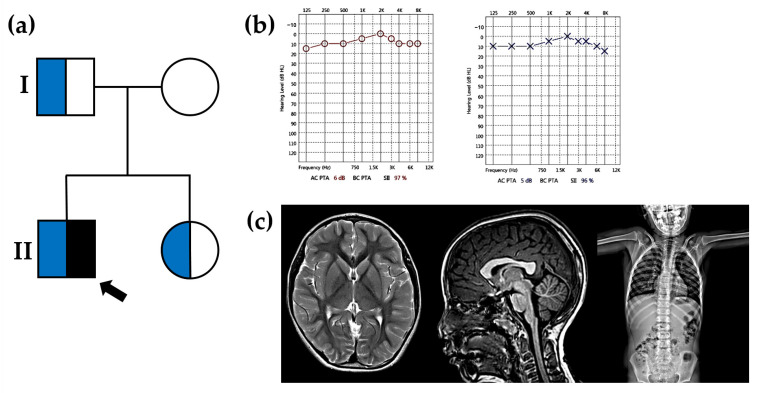
Pedigree and segregation analysis. (**a**) In generation I, the proband’s father has the trait. In generation II, the proband (indicated by black arrow) carrying a heterozygous *PTPN11* c.1492C > T/p.Arg498Trp variant in an autosomal dominant inheritance and his family members. Roman numerals (I and II) indicate the generation. Blue filled box indicates short stature and black filled box indicates intellectual disability. (**b**) Both hearing (right 6 dB and left 5 dB) was within the normal range. (**c**) Brain magnetic resonance imaging showed no structural abnormalities and appropriate myelination for the patient’s age, and spine X-ray revealed a normal structure with no apparent abnormalities.

The proband (II-1 in Figure 1a) is a 6-year-old boy with a short stature and ID, referred to the Department of Pediatric Neurology, Daejeon St. Mary’s hospital (Daejeon, Republic of Korea) for medical evaluation. He was born to nonconsanguineous, healthy Korean parents at 37 weeks of gestational age through normal vaginal delivery. At birth, his body weight was 2.5 kg (25th percentile), and his height was 46 cm (25th percentile). He is the first child, and the pregnancy was uneventful, with no family history of neurodevelopmental disorders. During the physical examination, no facial dysmorphism or skeletal abnormalities were observed, and there were no signs of lentigines or café-au-lait spots. The neonatal-to-toddler period was unremarkable; he began speaking at 12 months and walked independently at 13 months. However, issues with reading, writing, and social communication arose after entering kindergarten. His intellectual quotient (IQ) was estimated at 60 at 6 years old, indicating mild ID on the Wechsler Intelligence Scale for Children. Despite mild learning disabilities, he attended a preschool for normal children but received specialized education to enhance academic skills and social communication. At the age of 6, his height was 107.5 cm (5th percentile), and his body weight was 18.5 kg (10th percentile). His expected mid-parental height ranged from the 10th to the 20th percentile (169 cm), indicating a constitutional growth delay. Both hearing (right 6 dB and left 5 dB) and visual acuity were within the normal range (Figure 1b). Brain magnetic resonance imaging showed no structural abnormalities and appropriate myelination for the patient’s age, and spine X-ray revealed a normal structure with no apparent abnormalities (Figure 1c). Electrocardiography and echocardiogram results were also normal. Laboratory tests, including thyroid function, growth hormone, insulin-like growth factor (IGF)-I, IGF-binding protein 3, and metabolites, fell within the normal ranges. A wrist X-ray taken at the age of 6 indicated a bone age delayed to that of a 5-year-old child. Analysis of the peripheral blood smear revealed normal morphologies and counts, including white blood cells, platelets, and red blood cells. Clotting time tests, such as prothrombin time, activated partial thromboplastin time, and bleeding time, were performed and yielded results within the normal range. Urine analysis and a kidney sonogram yielded normal results without structural abnormalities. The skin condition, including color and texture, appeared to be within normal parameters. Conventional karyotyping revealed a normal male karyotype, and the *FMR1* (CGG)n triplet repeat test was negative. Chromosomal microarray analysis showed no copy number variations.

**Figure 2:**
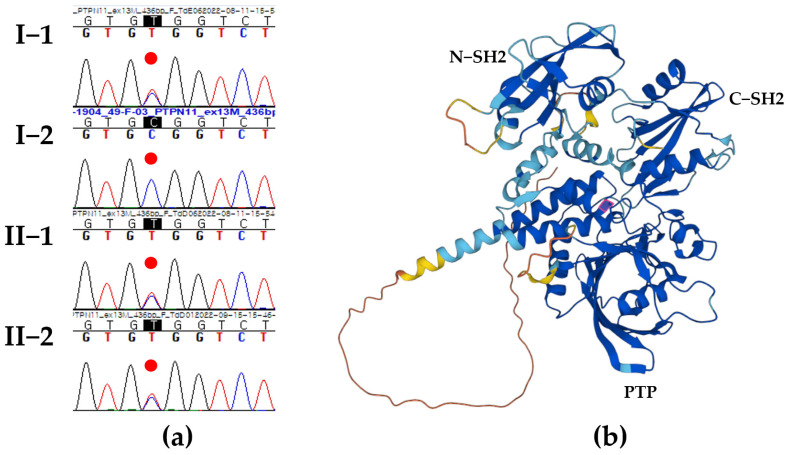
Segregation and protein structure analysis. (**a**) Sanger sequencing confirmed heterozygous *PTPN11* c.1492C>T/p.Arg498Trp variant of paternal origin in the proband (II-1) (Reference transcript ID: NM_002834.4). Each *PTPN11* variant is emphasized with the red dot. (**b**) Protein structure analysis using AlphaFold showed very high per-residue confidence scores (pLDDT) of 97.66 for PTPN11 p.Arg498 residue highlighted in pink box. Blue, sky blue, yellow, and orange color regions indicate pLDDT values greater than 90, between 70 and 90, between 50 and 70, and less than 50, respectively. Abbreviation: N−SH2, N-terminal Src-homology-2 domain; C−SH2, C-terminal Src-homology-2 domain, PTP, protein tyrosine phosphatase.

Then, the clinical exome sequencing was sequentially performed, and a heterozygous pathologic c.1492C > T/p.Arg498Trp of the *PTPN11* gene was the best candidate as the cause of the short stature and ID in the proband (Reference transcript ID: NM_002834.4). The clinical presentation of the proband was partially consistent with the *PTPN11* variant, indicative of Noonan syndrome 1 (OMIM # 163950), presenting with short stature, facial dysmorphism, and a wide spectrum of congenital heart defects. Sanger sequencing confirmed the segregation of the *PTPN11* p.Arg498Trp variant with the phenotype and established the autosomal dominant status of the heterozygous variant in both his father and sibling (Figure 2a). Protein structure analysis using AlphaFold (https://alphafold.ebi.ac.uk/; accessed on 16 March 2024) showed very high per-residue confidence scores (pLDDT) of 97.66 for the PTPN11 p.Arg498 residue highlighted in the pink box (Figure 2b).

### 3.2. The Proband’s Sibling

We assessed the proband’s sibling (II-2 in Figure 1a) for the first time when she was 4 years old. She was born at 37 weeks gestation, with a weight of 2450 g (25th percentile). At the age of 4, her height measured 90 cm (5–10th percentile), and her weight was 15 kg (5–10th percentile). Her expected mid-parental height is 156.5 cm (5–10th percentile). She exhibited normal intelligence, with an IQ of 100, and had no facial dysmorphism. An echocardiogram and electrocardiography were conducted, and the results were within the normal range. Visual acuity and auditory function appeared to be normal. Bone age assessment via wrist X-ray and hormonal level evaluations suggested a constitutional growth delay. Laboratory tests, including thyroid hormone levels and a skeletal survey, revealed no abnormalities. She attended a kindergarten for normal children and did not exhibit any medical problems.

### 3.3. The Proband’s Father

The proband’s father (I-1 in Figure 1a) experienced a completely normal development from birth to adolescence, with no reported learning difficulties. He successfully completed his college education without any abnormalities and is currently employed in the storage and delivery of goods. His height measured 163 cm (5th percentile), indicating familial growth delay. He maintains a good health without dysmorphism or deformities, and both echocardiogram and laboratory findings did not reveal any abnormalities.

## 4. Discussion

Many disorders present as autosomal dominant Mendelian traits and display a broad spectrum of phenotypes associated with factors such as penetrance, dominance, expressivity, and age-of-onset. Several hypotheses have been proposed to explain interfamilial and intra-familial phenotypic diversities in the context of the same gene variants. These include allelic variation, genetic backgrounds, ethnic origin, modifier genes, random mono-allelic expression, genetic compensation, environmental factors, and complex genetic and environmental interplay. Individual epigenetic variation is an important determinant of a range of clinical features [20]. Perturbation of a particular gene’s function in a pathway may alter the expression of other genes within the network, thereby influencing overall health [21]. Although many individuals with autosomal dominant NS have a de novo pathogenic variant, in 30%–75% of families, an affected parent is identified. NS/NSML can be diagnosed in a patient presenting with multiple lentigines and at least two other clinical features, such as cardiac problems, growth delay/short stature, facial dysmorphism, and pectus deformity [22]. Virtually all patients exhibit a dysmorphic face and multiple lentigines, with approximately 85% experiencing cardiac problems, including hypertrophic cardiomyopathy and pulmonary valve stenosis [23,24]. Growth delay leading to short stature is observed in more than 50% of patients, with heights falling below the 25th percentile for their age. Other clinical characteristics include sensorineural hearing loss (approximately 20%), ID (around 30%, typically in mild forms), and abnormal electrocardiography (seen in approximately 25% of cases) [25,26]. A literature review of NS patients with *PTPN11* p.Arg498Trp found that eight cases were genetically confirmed by genetic test [3,12,13,16,17,18]. Detailed clinical manifestations reported in individuals carrying the *PTPN11* p.Arg498Trp variant are summarized in Table 1.

**Figure 3:**
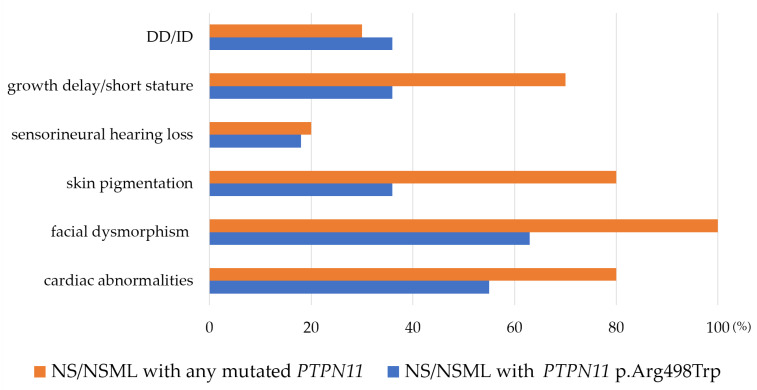
Different frequencies (%) of clinical features in Noonan syndrome (NS) or Noonan syndrome with multiple lentigines (NSML) carrying the *PTPN11* p.Arg498Trp variant compared to those with any other mutated *PTPN11*. DD/ID, developmental disability and intellectual disability.

In our family, the only common symptom observed was growth abnormalities (constituting constitutional growth delay and short stature), with other features such as cardiac problems and facial dysmorphism not co-existing. The proband exhibited mild ID and short stature without the typical characteristics of NS, which were inherited from his father, who had a normal intelligence and short stature. Among our cases and reported NS caused by the *PTPN11* p.Arg498Trp variant, cardiac abnormalities (6/11), facial dysmorphism (7/11), skin pigmentation (4/11), growth problems (5/11), and sensorineural hearing loss (2/11) have been observed. Compared to *PTPN11* variants in other locations [3,12,13,15,16,17,18,19], NS/NSML patients with the *PTPN11* p.Arg498Trp variant tend to exhibit relatively lower frequencies of skin pigmentation, facial dysmorphism, and cardiac abnormalities and mild symptoms, compared to those carrying any other mutated *PTPN11* (Figure 3), even though no statistically significant differences were observed, likely due to the considerable discrepancy in sample sizes when applying Pearson’s chi-squared or Fisher’s exact test.

Src-homology-2 (SH2) domain-containing protein-tyrosine phosphatase 2 (SHP2) is comprised of a catalytic protein-tyrosine phosphatase (PTP) domain that facilitates its association with signaling pathways [27]. Individual *PTPN11* variants exhibit specificity to each syndrome, with opposite effects on catalysis, yet all promote SHP2’s interaction with signaling partners. Mutations in the gain-of-function (GF) region of *PTPN11*, encoding the SHP2, are responsible for NS, primarily located at the interface between the N-terminal SH2 and PTP domains. These mutations alter residues involved in the autoinhibitory interaction, making the molecule prone to assuming an open conformation, thereby increasing the constitutive catalytic activity of the phosphatase [27]. On the other hand, reported dominant negative (DN) mutations of SHP2 are located solely within the PTP domain, near the substrate binding or catalytic sites of SHP2. These mutations have been shown to decrease phosphatase activity, resulting in NSML due to the DN effect [27]. *PTPN11* variants causing NS lead to SHP2’s GF and enhance RAS/MARK signaling [1].

On the other hand, *PTPN11* variants resulting in NSML lead to a reduction in the catalytic activity of the phosphate [22]. While NS and NSML share numerous clinical features, NSML has often been considered a variant of NS. However, the presence of ML as a definitive sign of NSML is infrequent in NS, and less penetrant NS features, such as a web-like neck, skeletal abnormalities, and hematological abnormalities, do not manifest in NSML [28]. Despite the similarity in phenotype, the precise differences in biochemical effects are not well-established. Yu et al. [29,30] reported that NSML-causing *PTPN11* variants not only result in reduced catalytic activity but also an increase in binding affinity/responsiveness, potentially contributing to overlapping clinical features. Particularly, members of the Spanish family carrying p.Thr357Met did not exhibit any apparent features fitting NSML or within the phenotypic spectrum of RASopathies. These findings reinforce the fact that the aberrant signal transduction resulting from *PTPN11* variants cannot be solely attributed to catalytic activity but necessitates enhanced binding of the phosphatase to signaling partners [31]. In our NS family with the *PTPN11* p.Arg498Trp variant, the proband exhibited ID and short stature without the typical characteristics of NS, and he inherited the variant from his father, who has normal intelligence. In another case report featuring the *PTPN11* p.Arg498Trp variant [32], a 9-year-old Korean boy exhibited a mild attention deficit and short stature, lacking typical manifestations such as facial dysmorphism, congenital heart disease, and chest deformities. Although his father also had a short stature, he did not exhibit symptoms suggestive of NS/NSML. Disparities based on gender, ethnicity, and environmental factors may potentially manifest inter-individually and within families. Moreover, in both the proband and his father in an American family carrying the *PTPN11* p.Arg498Trp variant, the proband, a 4-year-old boy with congenital profound deafness, underwent a comprehensive clinical re-evaluation, revealing minor craniofacial features that did not meet the diagnostic criteria for NS or NSML. The father, who was not available for clinical evaluation, denied having hearing loss and lentigines [13]. In a large-scale study of the clinical presentation and prevalence of lymphatic anomalies in patients with NS, patients with sporadic NS (16/40) have a high predisposition for developing lymphatic anomalies during life, compared to inherited NS (3/17, paternally inherited 1/6 and maternally inherited 2/11) [33].

Highlighting the phenotypic variability within families is crucial, especially when an affected family member has encountered severe cardiac or neurodevelopmental issues. The penetrance of NS in cases with *PTPN11* variants is nearly complete, with only one reported case of incomplete penetrance [34]. Variants were identified in 59% of individuals with familial NS, while only 37% of individuals with sporadic NS showed such defects. This statistically significant difference in *PTPN11* variant prevalence (*p* < 0.02) suggests that the additional gene or genes responsible for NS may contribute to incomplete penetrance or have more significant adverse effects on fertility than *PTPN11* [35]. Additionally, it is crucial to emphasize that the variant prevalence detected in any NS cohort is sensitive to its composition in terms of the relative abundance of sporadic and familial cases. In instances where a parent is affected with NS and bears a *PTPN11* variant, a sex-ratio bias is observed among offspring inheriting the defect [36]. This bias favors males by a factor of 2:1, and no sex-ratio distortion is noted among unaffected offspring. The available data suggest that this bias is linked to sex-specific developmental effects of *PTPN11* variants, favoring the survival of affected male embryos compared to female ones. Due to the limited number of familial cases of NS not associated with *PTPN11* variants, a systematic examination of sex-ratio bias in that genetic context has not been conducted.

## 5. Conclusions

Paternally inherited NS/NSML caused by a *PTPN11* p.Arg498Trp variant, including our cases, may exhibit relatively lower frequencies of abnormal features and mild symptoms. This could be ascribed to potential gene–gene interactions, gene–environment interactions, the gender and phenotype of the transmitting parent, or ethnic differences that influence the clinical phenotype. This study contributes to an understanding of the *PTPN11* p.Arg498Trp variant causing NS/NSML; further research is needed to comprehend the phenotypical variability, ranging from asymptomatic or very mild symptoms without key manifestations to typical NS/NSML.

## Figures and Tables

**Table 1:** Detailed clinical manifestations reported in individuals carrying *PTPN11* p.Arg498Trp variant.

References	This Report	This Report	This Report	Bademci et al. [13]	Edwards et al. [19]	Limongelli et al. [18]	Du-Thanh et al. [17]	Digilio et al. [15]	Kratz et al. [16]	Sarkozy et al. [12]	Sarkozy et al. [12]
Sex/Age at Dx	M/6 y	F/4 y	M/33 y	M/4 y	M/5 y	?/<1 y	F/39 y	?/<1 y	M/<1 y	F/2 y	F/34 y
Inheritance	Paternal	Paternal	n/a	Paternal	Sporadic	n/a	n/a	n/a	Sporadic	Maternal	n/a
Common features of NS											
Dysmorphic facial features	Neg	Neg	Neg	Pos (mild: hypertelorism, slightly low-set, posteriorly rotated ears)	Pos (mild: slightly macrocephaly, hypertelorism, mild ptosis, downslanting palpebral fissures, low-set and angulated ears)	Pos (full)	Pos (mild)	Pos	Neg	Pos (full)	Pos (full)
Cardiac abnormalities	Neg	Neg	Neg	Neg	Pos (PVS)	Pos (AR)	Neg	Pos (HCM)	Pos (HCM)	Pos (HCM)	Pos (HCM)
Growth delay/short stature	Pos (25th percentile)	Pos (5–10th percentile)	Pos (5th percentile)	Neg	Pos (<3th percentile)	Neg	Neg	Neg	n/a	n/a	n/a
Pectus deformity	Neg	Neg	Neg	Neg	Pos	Neg	Neg	n/a	Neg	n/a	n/a
Multiple Lentigines	Neg	Neg	Neg	Neg	Neg	Pos	Pos	Pos	n/a	Neg	Neg
Cryptorchidism	Neg	Neg	Neg	Neg	Pos	Neg	Neg	n/a	n/a	n/a	n/a
Infrequent features of NS											
Sensorineural hearing loss	Neg	Neg	Neg	Pos	Neg	Neg	Pos	Neg	Neg	Neg	Neg
Hematologic abnormalities	Neg	Neg	Neg	Neg	Neg	Neg	Neg	Neg	Pos (JMML)	Neg	Neg
Skeletal anomalies	Neg	Neg	Neg	Neg	n/a	Neg	Neg	n/a	Neg	n/a	n/a
Cafe-au-lait macules	Neg	Neg	Neg	Neg	Neg	Pos	Pos	Pos	n/a	Pos	Neg
Developmental delay/intellectual disability	Pos	Neg	Neg	Neg	Pos	Neg	Neg	n/a	n/a	Pos	Pos

Dx, diagnosis; M, male, F, female; y, year; PVS, pulmonic valve stenosis; HCM, hypertrophic cardiomyopathy, AR, aortic regurgitation; JMML, juvenile myelomonocytic leukemia; n/a, not available.

## Data Availability

Data are contained within the article.
